# Effect of novel polyethylene insert configurations on bone-implant micromotion and contact stresses in total ankle replacement prostheses: a finite element analysis

**DOI:** 10.3389/fbioe.2024.1371851

**Published:** 2024-04-18

**Authors:** Zhi Xu, Xiaonan Gong, Zhengyuan Hu, Ruixiang Bian, Ying Jin, Yuwan Li

**Affiliations:** ^1^ Department of Orthopedic, Zhangjiagang Fifth People’s Hospital, Zhangjiagang, Jiangsu, China; ^2^ Department of Orthopedic, Dongying People’s Hospital, Dongying, Shandong, China; ^3^ Department of Orthopedic, Jingxian Hospital, Jingxian, Anhui, China; ^4^ Department of Orthopedic, The Affiliated Hospital of Zunyi Medical University, Zunyi, Guizhou, China; ^5^ Department of Orthopedic, The First Affiliated Hospital, Zhejiang University School of Medicine, Hangzhou, Zhejiang, China

**Keywords:** total ankle replacement, artificial ankle, insert, elasticity improvement, finite element analysis

## Abstract

**Purpose:**

This study investigates the impact of elastic improvements to the artificial ankle joint insert on prosthesis biomechanics to reduce the risk of prosthesis loosening in TAR patients.

**Methods:**

CT data of the right ankle was collected from one elderly female volunteer. An original TAR model (Model A) was developed from CT images and the INBONE II implant system. The development of the new inserts adopts an elastic improvement design approach, where different geometric configurations of flexible layers are inserted into the traditional insert. The structure can be divided into continuous flexible layers and intermittent flexible layers. The flexible layers aim to improve the elasticity of the component by absorbing and dispersing more kinetic energy. The newly designed inserts are used to replace the original insert in Model A, resulting in the development of Models B-D. A finite element model of gait analysis was based by gait parameters. Discrepancies in micromotion and contact behaviour were analysed during the gait cycle, along with interface fretting and articular surface stress at 50% of the gait cycle.

**Results:**

In terms of micromotion, the improved elastic models showed reduced micromotion at the tibial-implant interfaces compared to the original model. The peak average micromotion decreased by 12.1%, 13.1%, and 14.5% in Models B, C, and D, respectively. The micromotion distribution also improved in the improved models, especially in Model D. Regarding contact areas, all models showed increased contact areas of articular surfaces with axial load, with Models B, C, and D increasing by 26.8%, 23.9%, and 24.4%, respectively. Contact stress on articular surfaces increased with axial load, reaching peak stress during the late stance phase. Models with continuous flexible layer designs exhibited lower stress levels. The insert and the talar prosthetic articular surfaces showed more uniform stress distribution in the improved models.

**Conclusion:**

Improving the elasticity of the insert can enhance component flexibility, absorb impact forces, reduce micromotion, and improve contact behavior. The design scheme of continuous flexible layers is more advantageous in transmitting and dispersing stress, providing reference value for insert improvement.

## 1 Introduction

Recently, as a treatment for ankle degeneration, total ankle replacement (TAR) has been increasingly used in clinical practice and has gradually become an alternative to arthrodesis (Terrell et al., 2013). Compared with those of arthrodesis, the technical advantages of the TAR include maintaining the mobility of the ankle, facilitating naturally representative gait behaviour, and maintaining the integrity of the subtalar joint, which is beneficial for the recovery of ankle function. Although TAR offers many advantages, it also exhibits clear disadvantages. The 5-year survival rate of TAR is 78%, and the 10-year survival rate drops to 62% (Henricson et al., 2007), which is lower than the average 5.5-year follow-up survival rate of 93% for ankle arthrodesis (Daniels et al., 2014).

Aseptic loosening is the main cause of TAR failure (Mahmoud et al., 2021). Because the ankle joint bears more load than the hip and knee joints (Thomas and Daniels, 2003), a higher load makes the ankle joint the most prone to stress overload in the lower limbs. A harsher mechanical environment than the hip or knee joint is more likely to cause sterile looseness between the implanted part of the artificial ankle joint and the surrounding bone. To improve the survival rate of TAR patients, cementless prosthetic implantation schemes have been tested in industry, and satisfactory results have been achieved (Gross et al., 2018). In these scenarios, the prosthesis is directly fixed by friction to avoid the possibility that the bone cement may damage the bone structure when filling the bone void, which can make the bone structure more complete and provide better bone structure support. These features are conducive to postoperative implantation.

In addition, the material selection of inserts is the key to successful implant design, and the selection of appropriate materials can prevent local stress overload (Figgie et al., 2014; Mondal and Ghosh, 2019a). At present, ultrahigh-molecular-weight polyethylene (UHMWPE) with high wear resistance and biological inertia is generally used as the material for manufacturing inserts. In addition, polyether ether ketone (PEEK) and carbon fibre-reinforced polyether ether ketone (CFR-PEEK), which are potential materials for artificial knee joint inserts, have also been described previously (Rankin et al., 2016; Koh et al., 2019a). Although PEEK and CFR-PEEK have favourable biomechanical properties (Abdullah et al., 2015), their wear performance is not as favourable as that of traditional UHMWPE in low consistent interfacial friction tests (Brockett et al., 2017); further research on biocompatibility and wear is needed (Koh et al., 2019a). The current fabrication of inserts essentially relies on a single homogeneous material (ASTM F2665–09 [S/OL]), and there are almost no reports on the compatibility of multiple materials involved in insert manufacturing. In addition, previous study has shown that the interface wear rate of artificial joint prostheses mainly depends on AP (anterior-posterior) and, IE (internal-external) kinematic behaviour (DesJardins et al., 2008). To reduce contact surface wear, previous researchers have focused on optimizing the radius of curvature of the prosthesis on the sagittal plane and coronal plane to improve the consistency of the prosthesis (Koh et al., 2019b). At present, there are almost no studies on the internal modification of inserts for the purpose of improving the mechanical properties of inserts. The author imagines whether a flexible material can be inserted into the noncontact interface layer of the insert to increase the overall elasticity, improve the flexibility of the insert and the ability to absorb loading, and provide favourable conditions for reducing bone–implant interface micromotion while improving the contact behaviour of articular surfaces.

Finite element analysis (FEA) is a numerical analysis method based on computer simulation, which has been used by many researchers to study ankle implants (Ozen et al., 2013; Wang et al., 2018). Finite element analysis is an important tool for studying micromotion (Sopher et al., 2017) and stress shielding (Saravana Kumar and George, 2017). Using FEA to develop TAR prostheses offers many advantages: (i) Predicting the micromotion of artificial ankle prosthesis-bone interfaces under different loads, thus evaluating the stability and durability of the prosthesis; (ii) Predicting the contact stress distribution at the prosthesis-bone interface and evaluating the mechanical coupling relationship between the prosthesis and bone tissue; and (iii) Finite element analysis can compare the differences in performance between different models under the premise of controlling variables, which is helpful for identifying the reasons for these differences, and contribute to conduct parameter studies.

In this study, a finite element model of the TAR was constructed by using an INBONE II implant system (Wright Medical Technology, United States of America). Novel configuration inserts for elastic improvement are designed based on the new concept of rigid-flexible-rigid stacking. We hypothesized that the improvement in elasticity of the inserts is related to the decrease in the micromotion of the bone-implant interface and the improvement in the contact behaviour of the articular surfaces. Elucidating this phenomenon may help to increase our understanding of the value of improving the elasticity of inserts in prolonging TAR cell survival. Therefore, this provides evidence for objectively evaluating the value of improving the elasticity of implants by comparing the differences in bone-implant micromotion and articular surface contact behaviour between different TAR models under the framework of the gait cycle.

## 2 Materials and methods

### 2.1 General information

A 66-year-old female volunteer, with a height of 163 cm and a weight of 73 kg, was recruited from the Joint Surgery Clinic of Zhangjiagang Fifth People’s Hospital in Jiangsu Province. This volunteer had no chronic underlying disease and no history of ankle trauma or surgery. Physical examination revealed that the movement of the right ankle was slightly limited, and X-ray examination revealed clear degenerative changes in the joint. The volunteers understood the purpose and details of this study and participated with informed consent. All methods in this study were performed in accordance with relevant regulations and guidelines. All protocols were approved by the ethics committee of Zhangjiagang Fifth People’s Hospital (L2023025).

### 2.2 CT image data acquisition

The volunteer was asked to maintain a supine position and a neutral position on the right ankle, and the right ankle was scanned with 64 rows of CT (GE Healthcare, United States of America). The scan ranged from 156 mm above the ankle space to 20 mm beyond the heel, covering the entire ankle and hindfoot. The scanning parameters were as follows: the slice thickness was 0.8 mm, and the bone threshold HU (Hounsfield Unit) was 226-1794. Acquisition matrix 512 × 512, pixel size 0.625 mm × 0.625 mm, field of view 400 mm × 400 mm. A total of 256 DICOM slices were obtained.

### 2.3 Intact model, implant modelling and TAR model construction

The CT data were extracted with Mimics 19.0 software (Materialise, Belgium), and a structural model of the normal foot and ankle joints was reconstructed based on the biological anatomy of the ankle. The geometric model file was imported into Geomagic wrap2017 software (Geomagic Company, United States of America) for subdivision, noise reduction, smoothing, accurate curved surface reconstruction and other processing steps to obtain a 3D solid model of bone, which was saved as an IGS file. To ensure the accuracy of the model, manual segmentation was conducted under the guidance of two experienced radiologists. The apparent density (ρ) and Young’s modulus (E) of each region were calculated by the HU value of the CT scan according to the Eqs 1, 2 (Tuncer et al., 2014):

$$\rho =0.0405+\left(9.18\times 10^{-4}\right)*HU$$ (1)

$$E=\left\{\begin{array}{c} 3.60\rho -0.14\;0<\rho \leq 0.1\\ 18.49\rho^{1.93}\;0.1<\rho \leq 0.37\\ 8.87\rho -0.57\;0.37<\rho \leq 1.5\\ 4.83\rho^{2.39}\;\rho >1.5 \end{array}\right.$$ (2)

**FIGURE 1:**
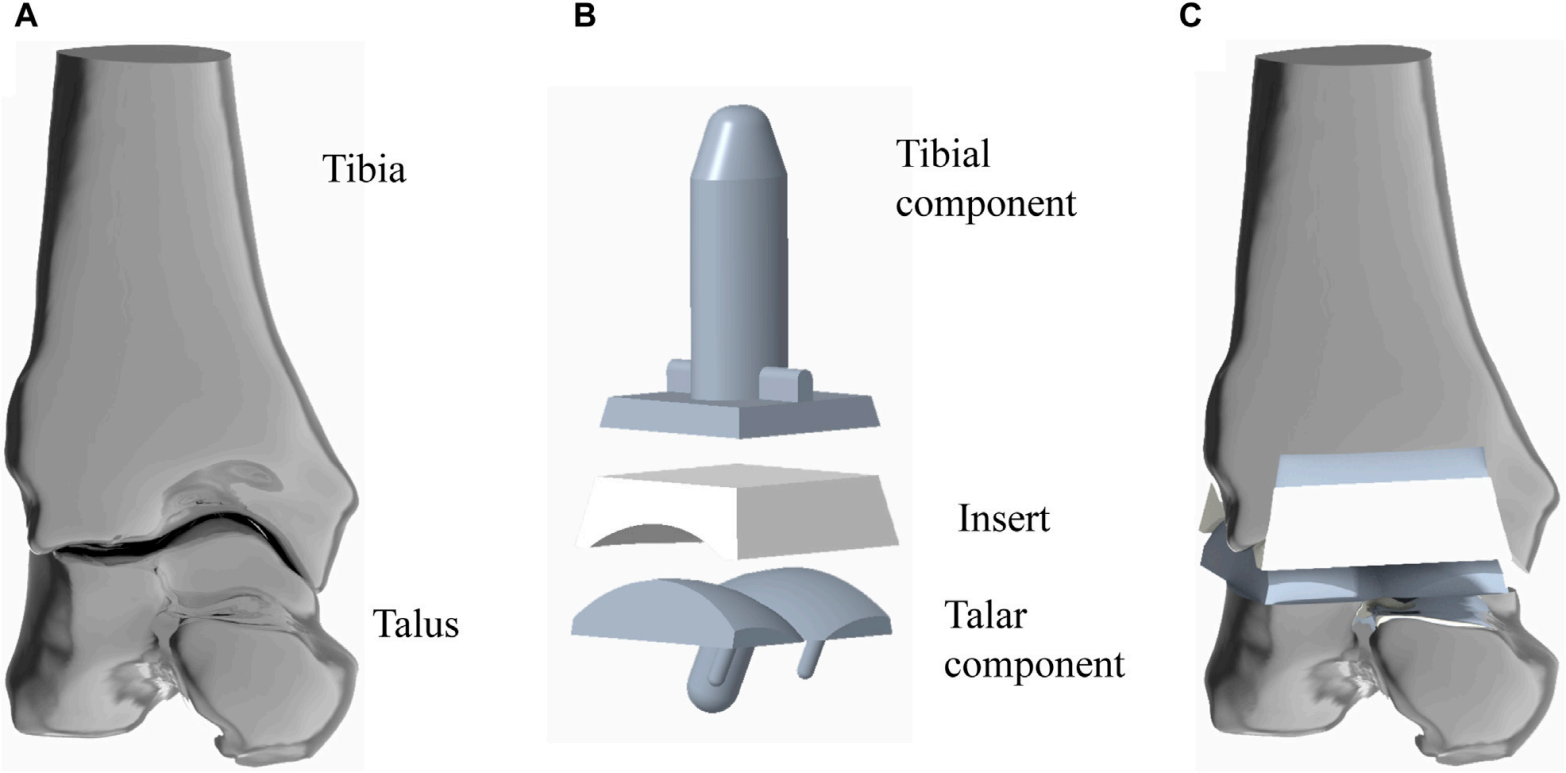
Simulated implantation of TAR prosthesis. **(A)** 3D model of ankle joint, the structure is the original model (Model A) in this study; **(B)** 3D model of TAR prosthesis; **(C)** assembled TAR ankle model.

The above IGS files were subsequently opened in turn with Creo6.0 software (PTC Company, United States of America), after which the intact model was assembled according to the experimental procedure (Figure 1A).

INBONE II is a reliable artificial ankle replacement system that is currently on the market in many countries. This study referred to the product manual provided by the dealer. Creo 6.0 software was used to model INBONE II, and the overall size of the implant model was adjusted according to the volunteer’s bone shape to match the implant shape to the bone shape. Model A was developed (Figure 1B).

**FIGURE 2:**
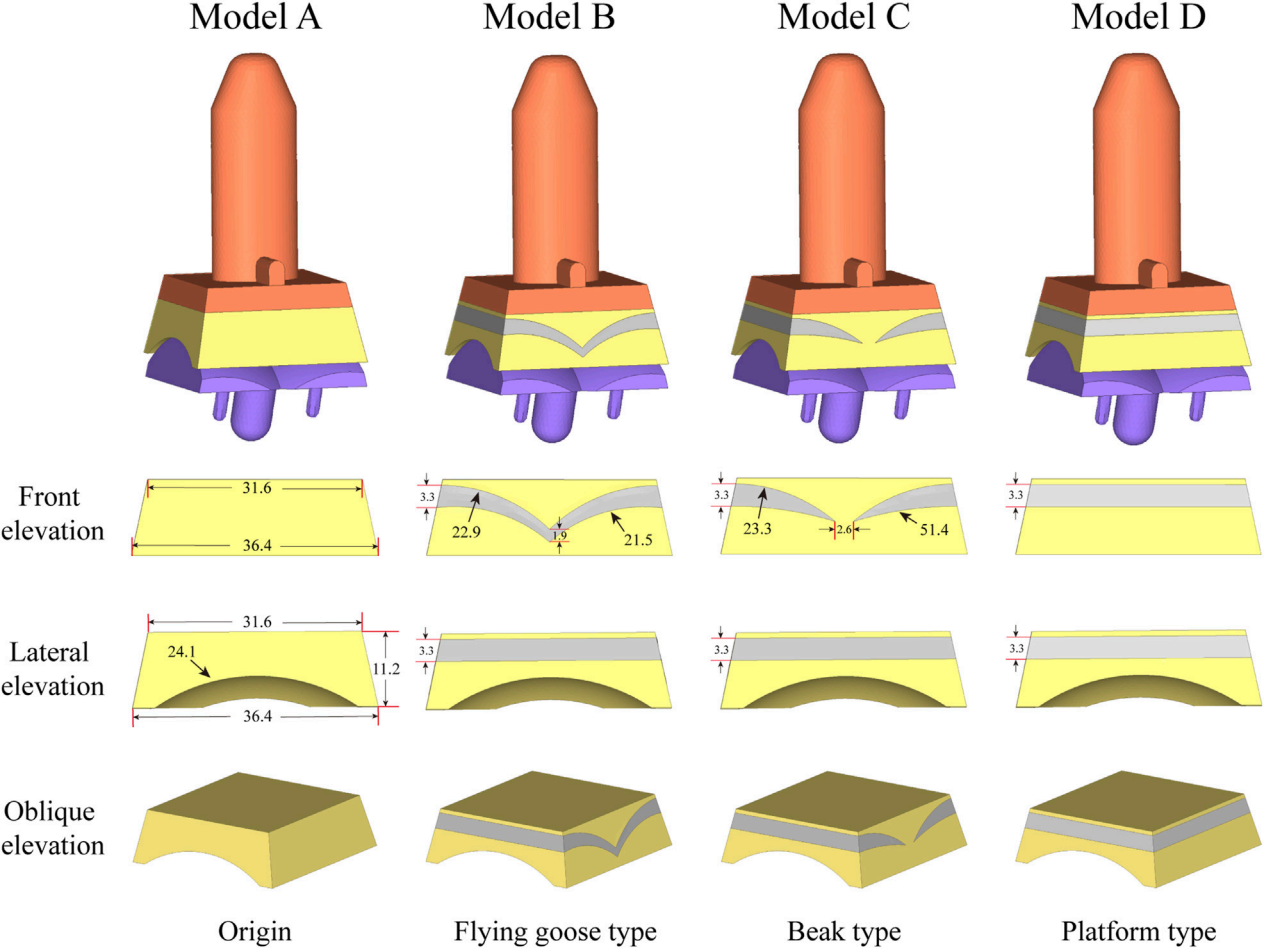
The model for optimizing the elasticity of the inserts used in the analysis is: Original TAR prosthesis; Flying goose type optimized TAR prosthesis; Beak type optimized TAR prosthesis; Platform type optimized TAR prosthesis.

To establish the TAR model, we performed osteotomy and prosthesis implantation according to the INBONE II operation guidelines provided by the supplier. First, the base plane was established to determine the position and angle of osteotomy, the tibia was first cut, and the talus was subsequently cut. During bone cutting, the medial and lateral tibias were maintained at an extroversion angle of approximately 60° towards the talus, and attention was given to protecting the lateral column of the tibia to maintain structural integrity and stability. The TAR model was constructed by selecting suitable tibia and talus prostheses and installing prostheses and UHMWPE inserts (Figure 1C). To ensure the rigor of the modelling, simulated surgery was conducted under the guidance and supervision of two senior orthopaedic surgeons. Figure 2 shows that on the basis of the original TAR (Model A), we replace the original insert with three types of novel configuration inserts, and we thus obtain three new TAR models (Model B-D, those will be introduced in detail in the next section) (Figure 2).

### 2.4 Improved elastic insert design

Figure 1B shows the basic design of model A, which consists of tibial/talar components and a insert, with the metal component fixed to the tibia and talus to provide support and stability. The articulating surfaces of the insert and talar component form a hinge joint to provide mobility to the joint. Models B-D replace new elastomeric enhanced inserts based on model A. Figure 2 shows the external designs of the three new inserts. Compared with Model A, Model B adopts a left-right symmetrical flexible material in the front view, and it is denoted the flying goose elastic improved insert. The purpose of this design scheme is to guide the load to be dispersed to the internal and external compartments through a continuous flexible layer symmetrical in the coronal plane to increase the overall flexibility and elasticity. Compared to Model B, Model C is also filled with curved flexible material; the difference is that the continuity of the original material is preserved in the 2.6 mm range near the central axis so that the optimized area has a bird’s beak shape. By maintaining intermittent flexible layers, soft areas are formed on both sides of the insert, which can absorb and disperse impact forces and reduce the contact stress of the joint surface. The middle part of the prosthesis, which is filled with traditional UHMWPE particles, provides overall support and strength. Compared to Model B, Model D adopted a platform design with a horizontal cross-section (losing the arc design) while retaining the continuity of the improved layer. Through the platform-shaped flexible layer and the connecting part of the traditional UHMWPE device, the pressure can be dispersed more evenly, increasing the energy absorption load of the component. Considering configuration, flying-goose-type and platform-type inserts are continuous flexible layer design schemes, while beak-type inserts are discontinuous flexible layer design schemes.

### 2.5 Mesh convergence test and material properties

**FIGURE 3:**
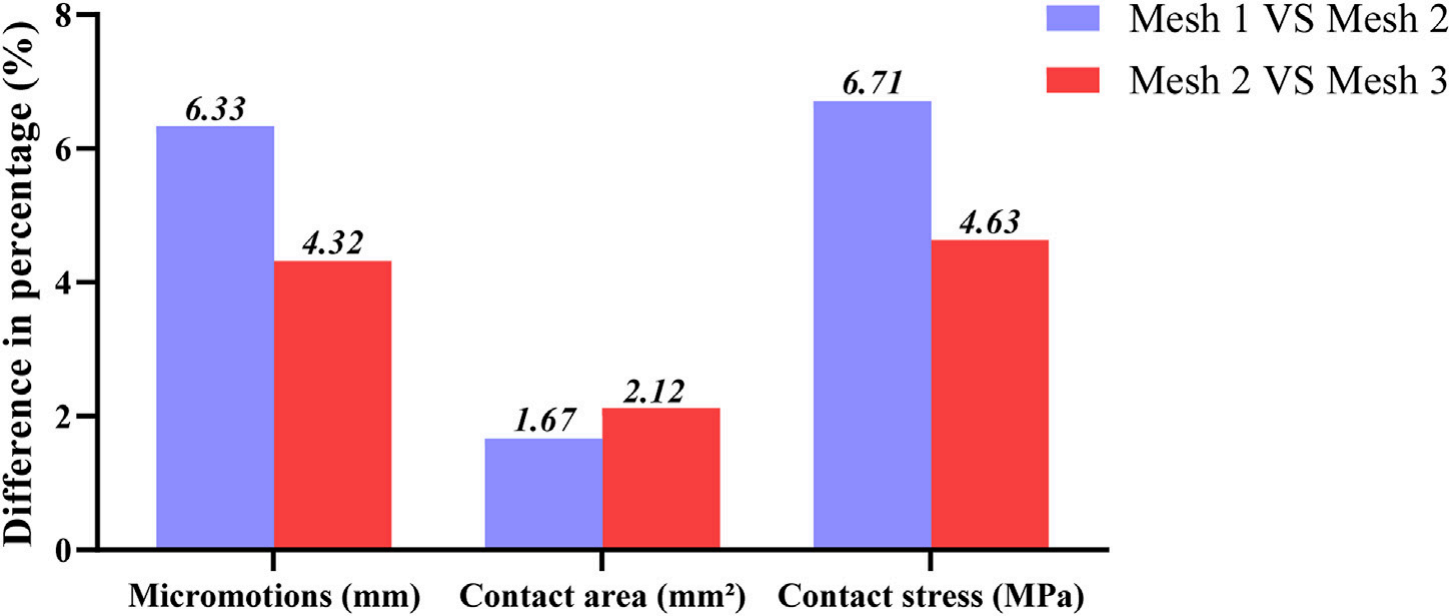
Percentage differences of max micromotion of the tibial component, articular surface contact area and max contact stress of the insert articular surface of TAR FEM models at three grid sizes. Change (%) = |Mesh 1/3 _value_–Mesh 2 _value_|/Mesh 2 _value_ * 100%.

The four TAR models developed by the above process were imported into Hypermesh 14.0 software (Altair, United States of America) as Parasolid assembly files. After geometry cleaning is complete, all the solid parts are meshed with four-node linear tetrahedral (C3D4) elements (Yu et al., 2022). To ensure that the selection of mesh size does not impact the predictions of the different models, we conducted a grid convergence study on the original TAR model and generated three corresponding grid resolutions according to the different grid sizes. Information describing the number of elements and nodes for each grid resolution is shown in Table 1. First, three TAR finite element models are generated according to the requirements of different mesh resolutions. These models can have different numbers of elements and nodes, achieved by varying the density or refinement of the grid. Then, load and boundary conditions were set for 50% of the gait cycle, and load and displacement constraints were applied to the tibia and talus to simulate the maximum load on the articular surfaces during the gait cycle. These boundary conditions are consistent across the three models. For each model with a different mesh size, the stress of each component was calculated using the FEA. Then, we selected the components of interest and testing metrics, namely maximum micromotion of the tibial component, articular surface contact area of the insert, and maximum contact stress, and compared the stress results of models with different mesh sizes. The value differences between different meshes are shown in Figure 3. The differences between parts of Mesh 2 and Mesh 3 are less than 5%. The evaluated parameters (bone-implant interface micromotion, contact area, and contact stress) have stabilized, and further reducing the mesh size has become insignificant. Mesh 2, with relatively fewer elements and nodes, demonstrates good convergence and can be utilized for further studies (Ayturk and Puttlitz, 2011). The grid components were saved as INP files and then imported into Abaqus6.14 (Dassault Company, France) finite element software for preprocessing. Detailed information on the number of units and nodes for each component is shown in Table 2. Bone and metal material components are simplified to linear elastic materials for assignment. According to the results of previous reports, each unit was assigned a separate material value according to the grey value formula. The insert was simulated as a nonlinear material, and the stress‒strain property was based on previously reported data (Miller et al., 2004; Yu et al., 2022). The details are listed in Table 3.

**TABLE 1:** Element and node numbers for three different mesh resolutions.

	Element number	Node number
Mesh 1	2,03,266	38,476
Mesh 2	4,84,469	98,226
Mesh 3	7,31,743	1,49,596

**TABLE 2:** Different model units and node information.

TAR model components	Element number	Node number
Tibia	1,88,544	36,472
Talus	1,18,010	22,291
Model A prostheses	1,77,915	39,463
Model B prostheses	2,06,072	43,919
Model C prostheses	2,04,609	43,729
Model D prostheses	2,07,985	44,232

**TABLE 3:** Material properties.

Components	Material	Elastic modulus (MPa)	Poisson’s ratio	References
Tibial component	CoCrMo	2,10,000	0.3	Mondal and Ghosh (2019b)
Talar component	CoCrMo	2,10,000	0.3	Mondal and Ghosh (2019a)
Insert	UHMWPE	1,174	0.46	Mondal and Ghosh (2019b)
Flexible insert	UHMWPE	556.92	0.46	Yu et al. (2022)
Distal Tibia	Bone	Based on CT greyscale	0.3	Tuncer et al., 2014; Zhang et al., 2023
talus	Bone	Based on CT greyscale	0.3	Tuncer et al., 2014; Zhang et al., 2023

### 2.6 Contact settings and boundary conditions

**FIGURE 4:**
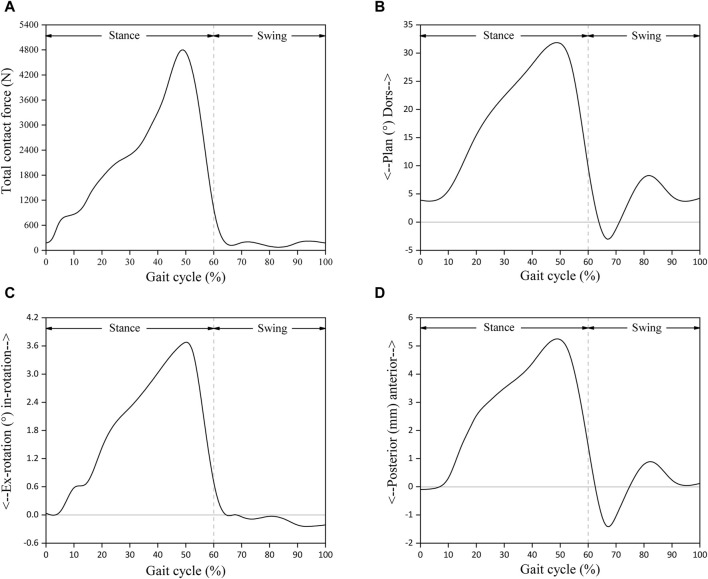
Input functions based on musculoskeletal multibody dynamics model: **(A)** Total joint contact force; **(B)** Foot dorsi-plantarflexion; **(C)** Tibial internal-external rotation; **(D)** Tibial anterior-posterior displacement. Reprinted with permission from Zhang et al., 2023.

**FIGURE 5:**
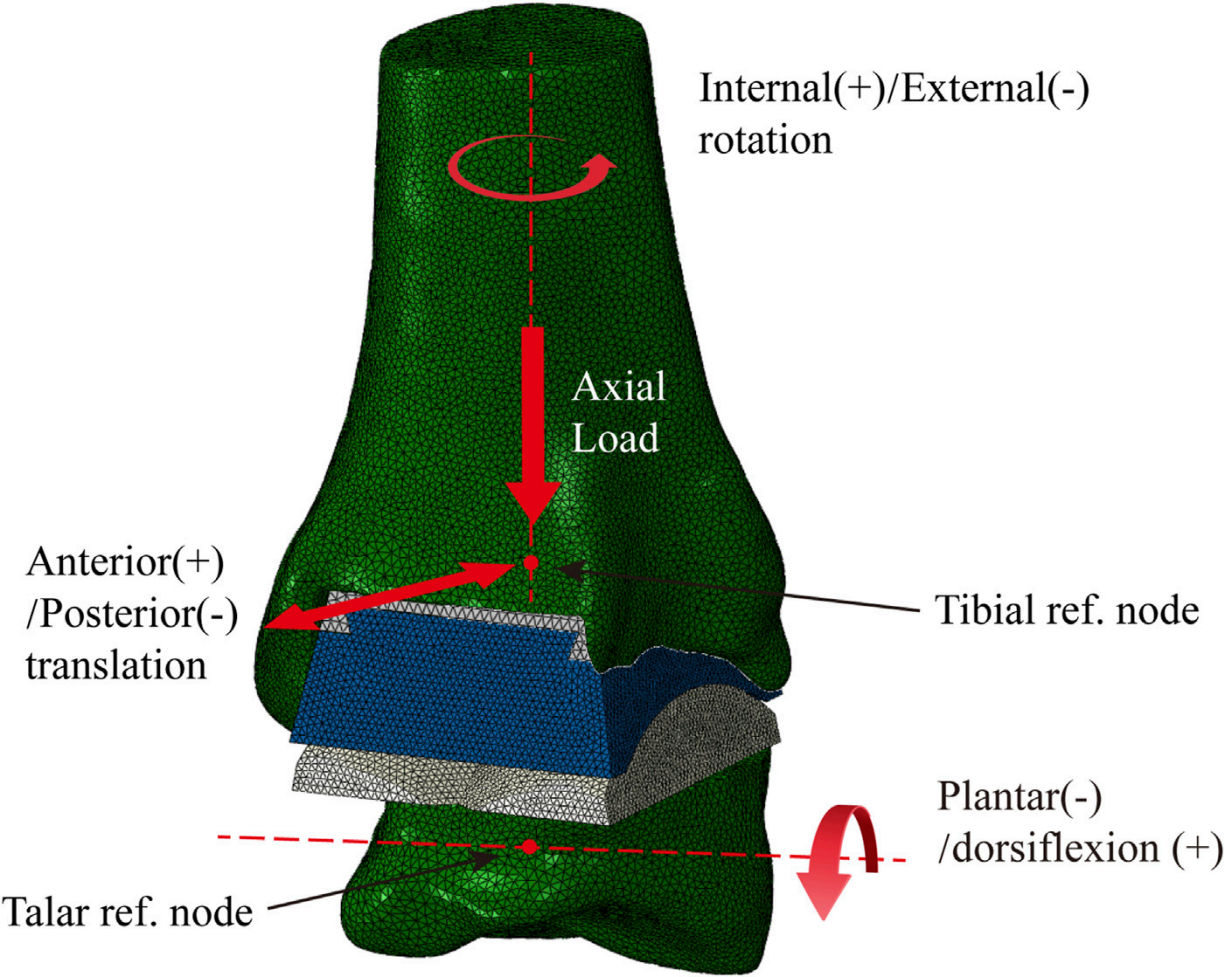
Finite element TAR model with defined boundary conditions.

All the computational models were subjected to contact setting after the assembly of Abaqus6.14. The bone-metal interfaces, which have more complex contact surfaces, were modeled using the explicit general contact algorithm, set as hard contact with a friction coefficient of μ = 0.5. For the insert-talar prothesis interface, which requires higher accuracy, the explicit contact pair algorithm was employed, set as hard contact with a friction coefficient of μ = 0.04, and the insert was in close contact with the tibial tray (Sopher et al., 2017; González et al., 2020). Zhang et al. (Zhang et al., 2020; Zhang et al., 2023) collected gait data from patients and used them as motor inputs for specific musculoskeletal models. The corresponding prediction of musculoskeletal model data for total ankle arthroplasty included dorsiflexion-metatarsal flexion, varus-valgus, internal-external rotation, anterior-posterior translation, lower-upper translation, internal and external translation, ankle contact force, muscle activation and tendon force. Compared to the data in other studies, the amplitude and trend of the predicted results are within a reasonable range. To simulate gait conditions, we referenced the research conducted by Zhang et al. (Zhang et al., 2020; Zhang et al., 2023) on gait experiments involving ankle implants and utilized ankle joint contact force and motion values based on the musculoskeletal (MSK) multibody dynamics (MBD) model used in that study. The entire gait cycle was divided into ten analysis steps, with gait parameters extracted at intervals of 10% and then subsequently used as the boundary and loading conditions for our TAR finite element models (Figure 4). Conforming to ISO 22622-2019, dorsiflexion-plantar flexion was applied to the reference node of the talus, which was coupled to the talus. AP translation and, I.E., rotation were applied on the reference node of the tibia, which was coupled to the distal tibia, and the reference node was defined as being on the axial force axis and 2 mm above the tibial tray. Axial load, displacement, and interexternal rotation were applied to the tibial reference point. All the other degrees of freedom of the tibia and talus were constrained (ISO, 2019; Zhang et al., 2023, Standard Specification for Total Ankle Replacement Prosthesis, 2014), and an intact TAR FEA model was developed (Figure 5).

### 2.7 Data processing and statistical analysis

In this study, the complete gait cycle was divided into 10 analysis steps using 10 as an integer multiple (10% was used as the interval), and the information from the last incremental step of each analysis step was extracted as the analysis data of the maximum bone-implant interface micromotion, articular surface contact area and maximum contact stress (The maximum value in the two items of interface micromotion and joint surface contact stress depends on the maximum value screening of high fretting/stress zone nodes, We collect data from all nodes on the interested contact surface, save the data in CSV format files, and use Excel software to read the CSV files. We use the MAX function based on a traversal algorithm to obtain the maximum value.). The variation curves for different models on different test items were obtained by using splines to connect these scattered points in the framework of the gait cycle. In order to further analyze the differences between different models in different test items, statistical analysis was conducted on the data of the 10 integer moments that different models participated in that constitute the gait cycle. SPSS 26.0 (SPSS, Inc., Chicago, IL) was used for the data analysis. The Shapiro‒Wilk test was performed to determine whether the data were normally distributed. The normally distributed data are expressed as the mean ± standard deviation. One-way analysis of variance (ANOVA) was used for the analysis of differences. Post hoc tests were performed using the LSD method when the differences were statistically significant. *p* < 0.05 was considered to indicate statistical significance.

## 3 Results

### 3.1 Bone-implant interface micromotion

**FIGURE 6:**
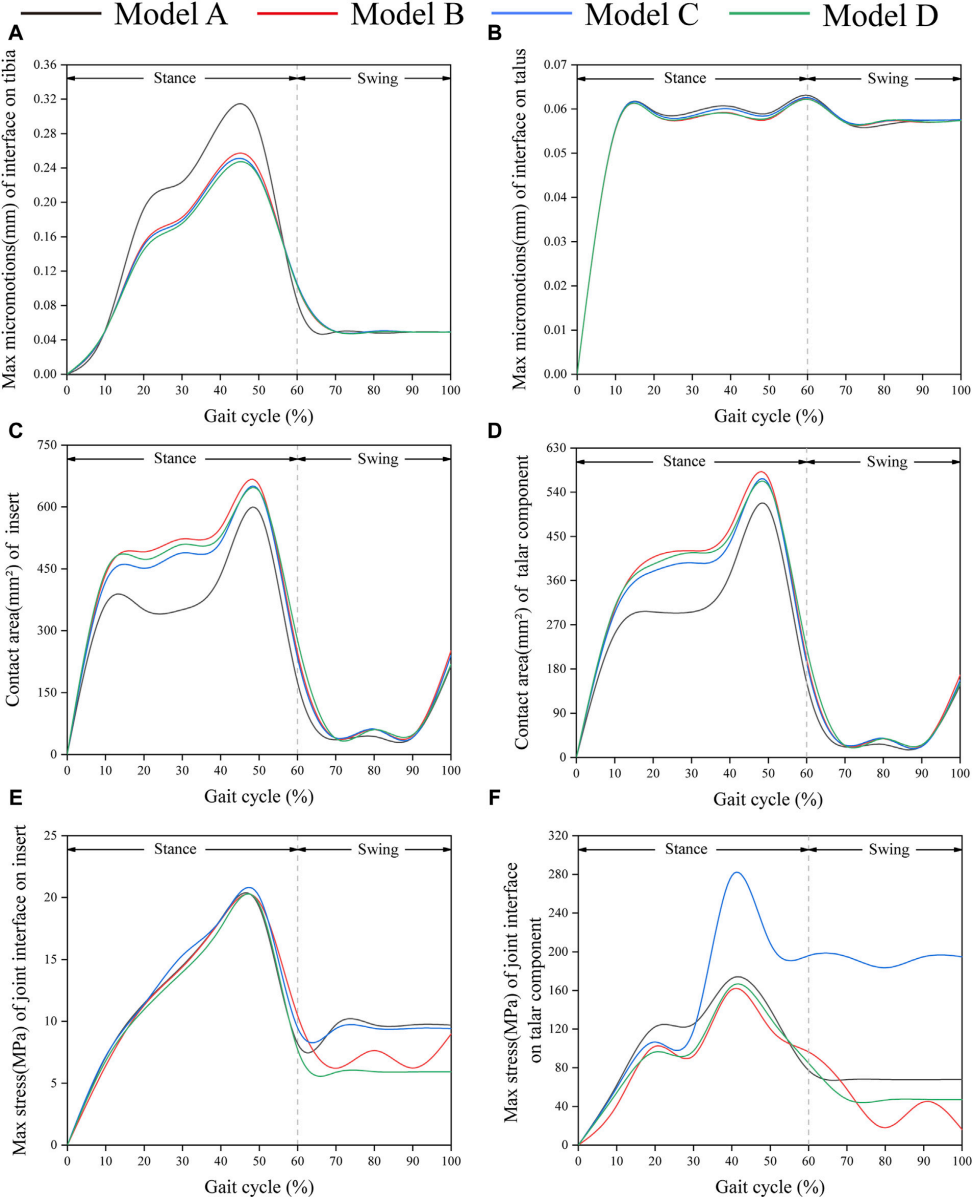
Comparison of biomechanical properties of gait in finite element models. **(A)** Maximum micromotion of the tibial end; **(B)** Maximum micromotion of talar end; **(C)** Contact area of articular surface of insert; **(D)** Contact area of articular surface of talar prosthesis; **(E)** Peak stress of articular surface of insert; **(F)** Peak stress of articular surface of talar prosthesis.

**FIGURE 7:**
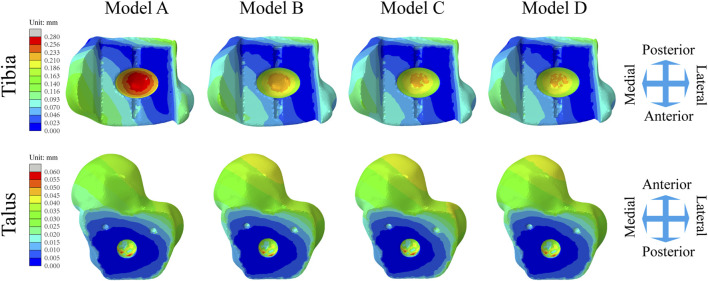
Distribution of the micromotion of the tibial/talar-implant interfaces at the instant of the peak micromotion for four TAR models at 50% of the gait cycle.

Figure 6A shows the changes in the maximum micromotion of the tibia-implant interfaces. The values of the four models all reached their peak in the later stage of the stance phase. The values of the three elastic improved TAR models are all less than those of the original model. Compared with Model A, the peak average micromotion of Model B, Model C and Model D interfaces decreased by 12.1%, 13.1% and 14.5%, respectively (Figure 9A). Figure 7 shows the micromotion distributions of the four TAR models at 50% of the gait cycle at the tibia-implant interfaces. A high micromotion scope can be observed at the dome of the tibia end in the original model (Model A) (red high displacement area aggregation can be observed). The red high micromotion area as predicted by the other three TAR models is significantly reduced in Model D, where the red high micromotion area is the least significant. The change trends of the maximum micromotion curves of the talus-implant interfaces for the different models are similar. With increasing load during the gait test, the micromotion of the interface gradually increased to 0.06 mm and reached its peak in the late stance phase (Figure 6B). Compared to those of Model A, the peak average values of the interface micromotion of Model B, Model C and Model D decreased by 0.9%, 0.2% and 0.8%, respectively (Figure 9B). According to the micromotion cloud map of the talus end, the high micromotion area was concentrated on the posterior surface of the central bone groove, and there was little difference in the red high micromotion area among the models (Figure 7).

### 3.2 Contact areas of the articular surfaces of the inserts and talar components

Figure 6C shows that the change in the contact area of the articular surfaces of the inserts in the four TAR models was similar during the gait cycle. With increasing axial load, the contact area of the articular surfaces of all the models increased and peaked at 50%. After entering the swing phase, the overall trend of the contact area decreased with decreasing axial stress. Compared with those of Model A, the average contact areas of Model B, Model C, and Model D increased by 26.8%, 23.9%, and 24.4%, respectively (Figure 9C). Similar results can be obtained when observing the trend of the contact area of the talar prostheses (Figure 6D), and the values of the elastic improved models are greater than those of the original model (Figure 9D). Therefore, improving the elasticity of the inserts has the advantage of increasing the contact area of the articular surfaces. Notably, flying-goose-type and platform-type configurations have more potential for applications than does the beak-type configuration.

### 3.3 Contact stress on the articular surfaces between inserts and talar components

**FIGURE 8:**
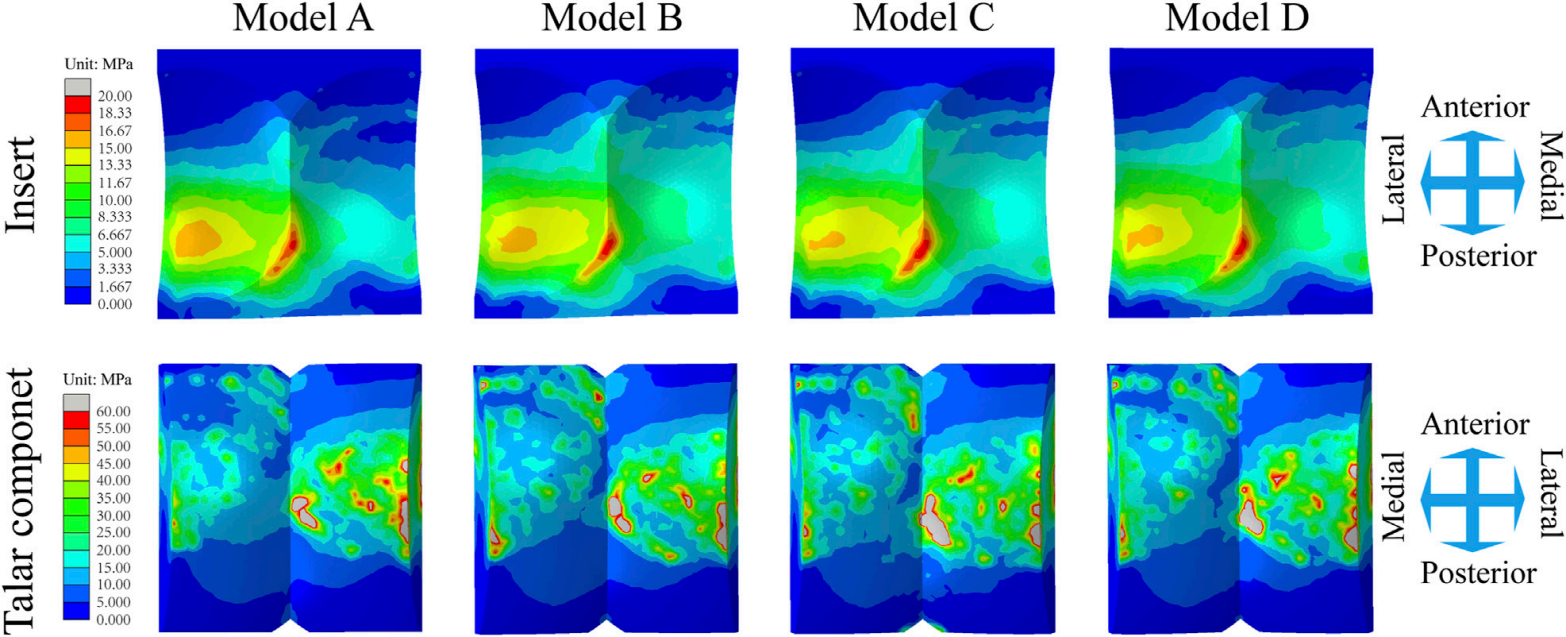
Distribution of the Mises stress of the inserts/talar components articular surfaces for four TAR models at 50% of the gait cycle.

**FIGURE 9:**
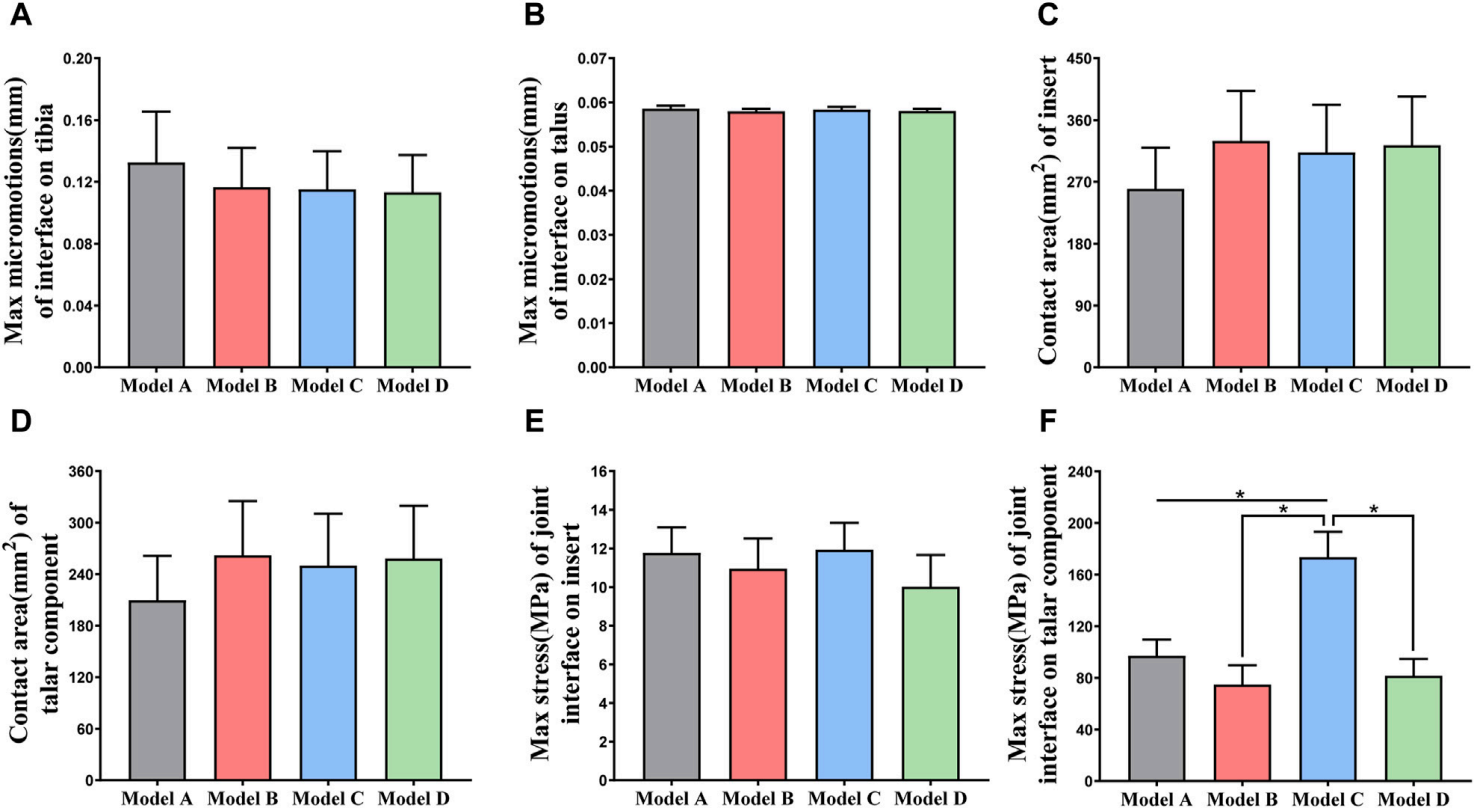
**(A, B)** Comparison of the maximum micromotion of the tibial/talar-implant interfaces of four models; **(C, D)** Comparison of the contact area of the articular surfaces of the TAR prostheses; **(E, F)** Comparison of the maximum stress of the articular surfaces of the inserts/talar prostheses. ^*^ *p* < 0.05 was used as cut off for bold significance.

Similarly, the maximum contact stress curves of the articular surfaces of the inserts of the four models exhibit similar trends, and the maximum surface stress increases with increasing axial load during the stance phase and reaches a peak during the gait period of 40%–50%, which is similar to the results of previous literature (Bischoff et al., 2019). The inserts of models B and D, utilizing a continuous flexible layer design scheme, demonstrate lower stress levels during the swing phase (Figure 6E). During this period, compared to model A, the mean maximum contact stress of the inserts for models B and D decreased by 6.9% and 14.9%, respectively, while model C increased by 1.5% (Figure 9E). Notably, the region of high stress on the medial articular surfaces of the improved inserts was less than that of the original model, which is of positive significance for improving contact behaviour (Figure 8). Figure 6F shows the trend of the maximum contact stress on the articular surfaces of the talus components, and the stress values of all the models increase to their highest values at the end of the stance phase. Compared to model A, the mean maximum contact stress of the talar prosthetic articular surface decreased by 22.8% and 15.9% for models B and D, respectively, while model B increased by 78.7% (Figure 9F). Cloud maps indicate a more uniform stress distribution on the improved models’ talar prosthetic articular surface compared to the original one, and model C exhibits continuous high-stress areas at the junction of the medial and lateral articular surfaces (Figure 8).

## 4 Discussion

This study used FEA to investigate various elastic design configurations for artificial ankle inserts. Different flexible layer structures are proposed to increase the absorption of gait impact and reduce prosthetic micromotion and contact stress on the joint surface. This innovative concept can provide guidance for further research on future artificial ankle insert design. We developed a TAR model system that includes the original INBONE II model and the elastic improvement model. Earlier studies directly reported the maximum contact stress of the insert and the maximum fretting range of the bone-implant interface under gait load. We thus compared the maximum tibia/talus-implant interface micromotion and insert maximum contact stress data of the four TAR models during the gait cycle with those reported in a previous study and found that the two sets of data were highly similar. Table 4 shows this detailed comparison. Finite element analysis of elastically improved inserts has not been performed previously. An important conclusion of the current study is that better elasticity is obtained by elastic-improved inserts, and the flexible UHMWPE device absorbs more of the gait load based on its improved elastic deformation characteristics, thus reducing the micromotion of the tibial and talar ends and improving the contact behaviour of the articular surfaces. Another important conclusion is that the area causing tibial prosthesis loosening is located at the top of the dome of the tibial groove (high micromotion area pointing to the red cloud diagram scope, as shown in Figure 6), and bone collapse has also been observed at the top of the tibial prostheses in related studies (Bolton-Maggs et al., 1985; Willegger et al., 2013).

**TABLE 4:** The maximum micromotion range of the bone-implant interfaces and the maximum contact stress of the inserts in the four models analyzed in this study were compared with previous studies.

References	Loading form	Micromotion (mm)	Max insert contact stress (MPa)
Martinelli et al. (2017)	Gait cycle load	–	19.8
Quevedo González et al. (2021)	Gait cycle load	0.0540–1.0760 for tibia	–
Zhang et al. (2022)	Gait cycle load	0.4042–0.4910 for tibia 0.0635–0.0646 for talus	31.62
Zhang et al. (2023)	Gait cycle load	0.1107–0.1905 for tibia	21.21
		0.0510–0.0691 for talus	
Present study	Gait cycle load	0.2312–0.2930 for tibia 0.0622–0.0631 for talus	20.08

Following the design idea of elastic improvement, we developed three new inserts based on UHMWPE material. Combined with the results of this study, the addition of flexible material to the liner of UHMWPE material can further improve its elasticity and flexibility, which has a certain value in reducing the micromotion of the bone-implant interface and reducing the contact stress of articular surfaces. The advantages of the improved elastic inserts are as follows: (i) A flexible UHMWPE device with a small elastic modulus can better adapt to changes in gait movement between the foot and ankle, thus reducing the micromotion of the bone-implant interface; and (ii) Filling the flexible UHMWPE device in the original insert can increase the flexibility characteristic so that, when the articular surface is under pressure, it can better disperse pressure and reduce maximum stress. The addition of flexible materials has also caused concern about the fatigue damage of novel inserts. Compared with traditional materials, flexible layers usually have low wear resistance and may be prone to wear and tear. This may shorten the life of the component, especially in the case of strong motion impact and repeated stress; the interfacial connection between the flexible layer and traditional UHMWPE may be affected by stress concentration and shear force between layers. If the interface connection is not strong, the flexible layer may fall off, delaminate or peel off, thus affecting the service life; in addition, the flexible layer may be affected by the introduction of design defects or material deterioration, resulting in deformation, deterioration or failure. This may reduce the function and life of the insert. Therefore, while flexible materials are added, it is necessary to comprehensively consider the elasticity, wear resistance, compression resistance and other properties of the materials to ensure the quality and reliability of artificial ankle bearings. In addition, in the manufacturing process, the addition of a flexible layer requires matching technology and equipment, and the bonding strength between the flexible layer and nonflexible layer is an important consideration. An insufficient bonding strength may lead to separation of the two materials, thus reducing the performance and life of the inserts. Ensuring the perfect combination of flexible layers and nonflexible layers, which poses a new challenge to the manufacture of improved inserts. Dreyer et al., 2023 indicated that the wear rate of fixed-insert was higher than rotating-platform insert, and the influence of insert structure on wear was greater than limb alignment. Favorable conformity can increase the contact area, reduce wear in the weight-bearing region. According to this text, the Flying goose type insert, Platform type insert, and Beak type insert may have different effects on joint surface wear. The Flying goose type insert and Platform type insert adopt a continuous flexible layer design, showing a reduction in micromotion and an increase in contact area in studies. This implies that these two designs may decrease the degree of joint surface wear, improve the stability and durability of the prosthesis. Additionally, Platform type insert also demonstrates even distribution of stress on the joint surface, with the lowest range of effective stress, which may help reduce joint surface wear. In contrast, the Beak type insert has an intermittent flexible layer design that may not be as effective in reducing micromotion and increasing contact area as the first two designs. Therefore, the Beak type insert may have a relatively weaker effect on reducing joint surface wear and improving prosthesis performance. In summary, the Flying goose type insert and Platform type insert may perform better in reducing joint surface wear and improving prosthesis stability, while the effectiveness of the Beak type insert may be relatively weak.

Due to the special anatomy and biomechanics of the ankle joint, the design of the ankle prosthesis cannot simulate the anatomy or biomechanics of the ankle joint, which limits the ability of the TAR to achieve successful artificial hip and knee replacement (Learmonth et al., 2007; Labek et al., 2011), which is mainly related to the anatomical characteristics of the ankle joint: (i) the contact area of the ankle joint is only 1/3 of that of the hip and knee joint, and the surface contact area under 500 N load is 350 mm^2^, 1,100 mm^2^ for the hip joint and 1,120 mm^2^ for the knee joint; (ii) the stress of the ankle joint is 5.5 times the body weight when standing, 3-4 times the knee joint weight, and 2-3 times the hip joint weight; and (iii) the joint matching degree of the ankle joint is very high, up to more than 95%, so that the articular surfaces of the ankle joint cover very thin cartilage, only 1.3 mm, while the knee joint is approximately 6–8 mm (Thomas and Daniels, 2003). In this study, we recorded the contact area of the articular surfaces throughout the whole process. At 10% of the gait cycle, the axial load is 866.5 N, and the maximum contact area of the insert is 363.9 mm^2^, which is close to the contact area of the tibial cartilage under physiological conditions. In this study, the elastic improvement inserts significantly increased the contact area of the joint surface, which may be attributed to the flexible layer absorbing the impact force and changing the motion adaptability. In addition, we also concluded that the flying goose-type insert and platform-type insert performed better at increasing the joint surface contact area, while the beak-type insert had a relatively poor effect. This difference may be related to the geometric configuration of the flexible layer. The flexible layers of the flying goose-type insert and platform-type insert are continuous, which signifies that under a gait load, the continuous flexible layers can provide more uniform and continuous support, which can better adapt to movement changes and effectively disperse stress and bring the insert into better contact with the joint surface. In contrast, the flexible layer of the beak-type insert is discontinuous, which may result in less uniform load sharing during the gait cycle. Because the central part of the beak-type insert is made of traditional UHMWPE particles, the insert has an (in-)elastic hinge due to material inhomogeneity, when the axial load increases, the load in the central part of the insert cannot be dispersed, preventing favourable adaptation to gait changes, stress mainly conducts along the traditional UHMWPE, leading to stress concentration at the junction of the medial and lateral articular surfaces.

There has been much prior research on prosthesis failure. Compared to artificial hip and knee joints, which are more prone to wear, fatigue and corrosion, the main failure factor of artificial ankle joints is aseptic loosening of prostheses (Messellek et al., 2020; Feyzi et al., 2021). To improve the fixation effect of prostheses, experts have advocated the use of noncement implantation in recent years (Gross et al., 2018), which relies on friction fixation between the surfaces of the implant and the bone groove, which overcomes local complications caused by bone cement and thus creates a more compatible environment for inwards bone growth. The initial stability of different types of implants can be evaluated with the interface micromotion index. This approach helps to reduce the risk of implant loosening (Angthong et al., 2013; Brunner et al., 2013; McInnes et al., 2014). In this study, we tested the interface micromotion of four TAR models and found that the overall level of micromotion at the tibial ends was greater than that at the talar ends. Combined with the numerical and cloud image results, the effect of improving tibial interface micromotion is ranked (from lowest to highest) as follows: Model D, Model C, Model B, and Model A.

Observing the interface micromotion situation of the talus, although the values of the improved models were still smaller than those of the original model, the improvement was not clear in terms of the numerical value. Compared with that of the original model, the maximum micromotion mean value of the tibia-metal prosthesis interface in the improved model decreased by 16.1–19.3 μm, while that of the improved model talus-metal prosthesis interface decreased by 0.1–0.6 μm. After the insertion improved in terms of elasticity, the benefit of micromotion reduction at the tibial ends was greater than that at the talar ends. A possible explanation for this result is that the normal direction of micromotion at the end of the tibia is the same as the direction of the load, and the angular fixation of three oblique nails at the end of the talus can effectively prevent the micromotion caused by axial stress.

In addition, we compared the contact and stress behaviours of the different inserts. The core question investigated in this study is whether the continuity of the UHMWPE flexible layer will improve contact behaviour. An optimistic conclusion, drawn from the results shown in Figure 8A, C, D, E, is that flying goose- and platform-type inserts (continuous flexible layer) achieve lower micromotion and articular surface contact stress and a larger contact area than the beak type insert (intermittent flexible layer). We also discuss the difference in mechanical properties between the continuous flexible layer scheme and discontinuous flexible layer scheme, and we propose that the advantages of the former are as follows: (i) The full layer through flexible layer inserts has a larger contact area and better deformation capacity. With these insert shapes, the flexible layer completely penetrates the whole component and can effectively absorb and disperse the impact energy. On the other hand, a flexible layer is only inserted into the beak-type gasket on both sides, and the material in the original gasket is maintained in the middle, higher vertical stiffness in the centre, resulting in a smaller contact area and poor deformation capacity, which limits its ability to absorb energy. (ii) The full-layer penetrating flexible layer inserts can quickly transfer the impact energy to the whole flexible layer and disperse the energy in the component. This ability to disperse energy helps to reduce the transfer of impact force to the prosthesis and reduce the fretting of the prosthesis. Because the middle part of the beak-type insert is still the original material, it cannot fully exert the energy dispersion function of the flexible layer, which limits its anti-seismic effect. The difference in design between two continuous flexible layers is further discussed. The platform type design is conducive to the uniform transfer of load in the flexible layer, so it has more advantages in reducing the fretting of the prosthesis and improving contact behaviour, but the flying goose type design is impressive in reducing the peak stress of the insert during the gait cycle. The geometry of curved surfaces may create favourable conditions for the dispersion of axial loads. In brief, the two continuous flexible layer designs exhibit favourable mechanical properties. Considering that elastically improved inserts have favourable design potential, regulating the thickness of flexible layers with different configurations may have a positive impact on their mechanical properties. We can assume that a thicker flexible layer has better seismic resistance. The reason for this difference is as follows: the thicker the flexible layer is, the greater the deformation space and elastic capacity of the insert, which can absorb more kinetic energy and provide a greater cushioning effect, at the same time reducing the load transferred to the artificial ankle prostheses, thus reducing the micromotion of the prostheses. This further inspires our confidence in improving artificial ankle inserts.

It is also an exciting topics to discuss the application scenarios of the improved inserts. Severe ankle joint diseases include advanced degenerative arthritis, advanced rheumatoid arthritis, severe traumatic arthritis, etc. When conservative treatment fails to control the continuous deterioration of the condition, leading to pain and functional impairment, it may be necessary to consider ankle joint replacement surgery. Traditional ankle joint liners may not provide enough compliance and comfort, leading to pain and functional impairment. By inserting a flexible layer in the insert, the pressure on the joint and micromotion at the interface can be effectively reduced, improving the joint’s motion mechanism, thereby reducing pain. Patients may find a smoother gait and wider range of joint motion in their daily lives, thus improving their quality of life. For sports enthusiasts or professional athletes, undergoing ankle joint replacement with improved inserts may help them recover to a state closer to normal ankle joint function, improving their athletic performance and allowing them to better participate in various sports activities. Customized improved inserts tailored to the specific needs of patients can make treatment more personalized. This personalized design can be adjusted based on factors such as the patient’s age and activity level, providing a treatment plan that better meets the patient’s needs.

Studying the biomechanical relationship between the ankle and knee joints, revealing the potential of improved insert design to enhance lower limb joint function and protection, can provide new insights and theoretical support for clinical treatment of joint replacement surgery. Combining previous scholars’ simulation and discussion on joint cartilage and ligament injury (Adouni et al., 2019; Adouni et al., 2020; Adouni et al., 2023), the present study suggests that the impact of the artificial ankle joint improved insert on the knee joint may be multifaceted. Firstly, by incorporating more flexible and elastic improved insert designs, it may help reduce the impact and pressure on the ankle joint, thereby lowering the overall burden on the lower limb joints. This load reduction effect could potentially transfer to the knee joint, decreasing the pressure and stress on knee cartilage and ligaments, consequently reducing the risk of cartilage and ligament injuries. The application of a flexible layer might aid in reducing joint micromotion, improving contact behavior, enhancing stability of the prosthesis during movement, further protecting the knee joint from injuries. Additionally, the improved insert could enhance ankle stability and motion control, reducing adverse effects of abnormal ankle movements on the knee joint. A stable ankle joint can facilitate smooth operation of the entire lower limb kinetic chain, minimizing the additional pressure on knee soft tissues from abnormal movement patterns and postures, thus reducing the risk of soft tissue injuries. Therefore, the improved insert for artificial ankle joints may play a beneficial role in protecting knee cartilage and ligaments, reducing injury risks, and enhancing overall stability of lower limb joints.

However, this study has several limitations. First, the CT image data used for FEA were obtained from an elderly woman with ankle degeneration. Whether the conclusion is applicable to explain the efficacy of surgical intervention in patients with different ankle joint degenerations remains to be further observed and confirmed. Second, finite element analysis typically requires certain simplifications and presets to be applied to characterize the artificial ankle joints. We ignore the effects of inertial loading and damping, however materials damping and component damping would affect the vibration response and force transmission of the joint model. Proper damping design can reduce joint surface oscillation, decrease fluctuations in contact stress, and potentially reduce the risk of prosthetic loosening. In brief, the ability of FEA to predict the stress results of models is limited by the experimental object, the quality and sensitivity of the mesh, the load conditions and other factors, which cannot replace physical experiments. Therefore, the conclusions of this study should be carefully interpreted. In view of the limitations of this study, we plan to manufacture the designed TAR prosthesis and complete laboratory biomechanical tests to further verify the feasibility and effectiveness of elastically improved inserts.

## 5 Conclusion

This study showed that by adding continuous flexible layer to the original insert, the overall elasticity can increase, which is beneficial for absorbing the force of the joint and improving the interface contact behaviour. Compared to those of the original model, the bone-implant interface micromotion of the elastic improved TAR models was lower, and the contact area was larger. Additionally, variations in geometry result in differences in the mechanical performance of three types of elasticized improved inserts. Based on evaluations of interface micromotion and articular surface contact behavior, we believe that the design featuring a continuous flexible layer is the preferable choice.

## Data Availability

The raw data supporting the conclusion of this article will be made available by the authors, without undue reservation.

## Abbreviations

TAR, Total ankle replacement; FEA, Finite element analysis; UHMWPE, Ultra-high molecular weight polyethylene; PEEK, Polyether ether ketone; CFR-PEEK, Carbon fiber reinforced polyether ether ketone; DICOM, Digital Imaging and Communications in Medicine format; ISO, International Organization for Standardization.
